# The epistemological implications of species extinction: An overview

**DOI:** 10.1007/s13280-025-02230-9

**Published:** 2025-08-09

**Authors:** Johannes M. Luetz

**Affiliations:** 1 Graduate Research School, Alphacrucis University College, Brisbane, QLD 4102 Australia; 2 School of Law and Society, The University of the Sunshine Coast, Maroochydore, QLD 4556 Australia; 3 School of Social Sciences, The University of New South Wales, Sydney, NSW 2052 Australia

**Keywords:** Biodiversity conservation, Cultural epistemologies, Epistemological consequences, Scientific epistemologies, Species extinction, Spiritual epistemologies

## Abstract

The epistemological implications of species extinction remain widely underexplored. This theoretical paper addresses this gap by examining the linkages between extinction and epistemology across three interconnected dimensions: science, community, and metaphysics. Highlighting the profound and irreversible losses of extinction, this article argues for conservation as a vital strategy to preserve knowledge, community, and existential meaning.

## The benefits of examining the epistemological implications of species extinction

The epistemological consequences of extinction remain under-theorised in the literature and benefit from being conceptualised in a manner that is scientific, inclusive, and holistic. A review of the limited available scholarship indicates a substantial knowledge gap in the literature and points to fertile research opportunities (Heise 2016; Schuster 2023; Tanswell 2024). While the ethics of species extinctions have been documented in the literature (Wienhues et al. 2023), Tanswell (2024) has summarised that “the concept of extinction has itself received surprisingly little attention from philosophers” (p. 205). Set against this literary background, this conceptual paper examines the epistemological implications of species extinction in the areas of science, community, and metaphysics, thus contributing theoretical perspectives on biodiversity loss that transcend purely ecological concerns. It is premised on a consilient view of science and the humanities wherein these paradigms are conceived as complementary rather than competitive. Crucially, recognising and conceptualising the multifaceted epistemological consequences of extinction can invigorate conservation strategies and foster a deeper understanding of—and connection with—the natural world.

## Extinction: Perspectives, statistics, and prospects

This section introduces extinction as a word, concept, and contemporary phenomenon and sets the scene for discussion of its diverse epistemological implications. The word “extinction” originates from the Latin term *extinctio*, which stems from *extinguere*, meaning “to put out” or “to quench”, particularly in reference to fire, light, or life (McKean 2005, p. 597). Figuratively speaking, extinction means “putting out” the light of existence. This article presents the progressive extinction of species as a gradual “dimming” of the light of essential knowledge in areas of science, community, and metaphysics. It adopts an interfaith positionality that embraces a worldview-inclusive approach to matters of faith (Luetz et al. 2023).

In biological terms, extinction refers to the complete disappearance of a species, group, or population from its natural environment—or the earth as a whole (Barnosky et al. 2011). Extinction occurs when a species can no longer survive and reproduce in its natural habitat, often due to changes in environmental conditions, habitat loss, overexploitation, and/or competition from other species (Kolbert 2014). According to Purvis et al. (2000), “extinction is conceptually simple—a species is extinct when its last member has died” (p. 1124). As such, “in the life of any species, extinction is the final evolutionary process” (Purvis et al. 2000, p. 1123).

Science can help reveal the scale and rate of contemporary extinction. In 2017, 15 364 scientist signatories from 184 countries issued the so-called World Scientists’ Warning to Humanity: “humanity has … unleashed a mass extinction event, the sixth in roughly 540 million years, wherein many current life forms could be annihilated or at least committed to extinction by the end of this century” (Ripple et al. 2017, p. 1026). Two years later, the United Nations (UN 2019) warned that “Humans are driving one million species to extinction” (Tollefson 2019, p. 171) in what is now acknowledged among scientists as the sixth and most ruinous extinction event in the earth’s history (IPBES 2019; Kolbert 2014). Tollefson (2019) describes the nature-based assessment that led to this warning (IPBES 2019) as “the most comprehensive report yet on the state of global ecosystems” (p. 171). Drawing on the support of 132 signatory governments, the analysis “distils findings from nearly 15 000 studies and government reports, integrating information from the natural and social sciences, Indigenous peoples and traditional agricultural communities” (Tollefson 2019, p. 171).

Despite this vast body of research, the exact number of species that have verifiably gone extinct during the current sixth mass extinction is challenging to determine, including on account of incomplete records, the scientific difficulties of proving a negative, and the ongoing, interconnected, and accelerating nature of relevant processes (Strona and Bradshaw 2022; Tudge 2000; UN 2019). Scientists acknowledge this outright: “The precise number of recent extinctions is impossible to know” (Ceballos and Ehrlich 2023, p. 1). This picture is further complicated by the insidious rise of extinction denialism, driven by “politically well-connected and well-funded antagonists” (Lees et al. 2020, p. 1440). Nevertheless, despite these and other challenges, scientific studies have investigated the building magnitude of human-caused biodiversity losses, establishing the extinction of 562 bird species (Matthews et al. 2024, p. 2), 543 vertebrate species (Ceballos et al. 2020, p. 13 599), and a total 73 *genera*^1^ of species lost since 1500 (Ceballos and Ehrlich 2023), among numerous other assemblages of statistics and estimates (IPBES 2019; UN 2019; IUCN Red List 2024).

Placing these figures within the context of geological timescales highlights that current extinction levels vastly exceed the natural so-called “background rate” that might be expected in the absence of human interference (De Vos et al. 2015). Human activities have significantly increased species extinction rates “tens to hundreds of times higher than the average across the past ten million years” (Tollefson 2019, p. 171). For example, Ceballos and Ehrlich (2023) found that extinction rates among *genera* are “35 times higher than expected background rates prevailing in the last million years under the absence of human impacts” (p. 1). Zooming in on vertebrates, Ceballos et al. (2017) have documented the extinction of 200 vertebrate species in the last 100 years, representing the loss of about two species per year. The authors note that according to the normal background rate prevailing in the last two million years, “the 200 vertebrate species losses would have taken not a century, but up to 10 000 years to disappear” (p. E6089). Similarly, Matthews et al. (2024) have shown that the human-caused extinction of 562 bird species is tantamount in significance to the loss of “3 billion years of unique evolutionary history” (p. 2). In synthesis, humans “have increased species extinction rates by orders of magnitude compared with the background extinction rate” (Matthews et al. 2024, p. 1).

Significantly, Ceballos et al. (2020) purport that “extinction cascades” (p. 13 600), meaning that the disappearance of one species sends ripple effects to “its corresponding local food web” (Strona and Bradshaw 2022, p. 5). This can create a domino effect, whereby the extinction of one species may set off a chain reaction of associated co-extinctions (Anderson et al. 2011; Strona and Bradshaw 2022). Consequently, the combined net effect of individual species extinctions is considerably greater than the sum of constituent parts—the loss of one species may disrupt vital ecological networks and send cascading consequences across entire ecosystems (Anderson et al. 2011; Ripple et al. 2017; Strona and Bradshaw 2022; Ceballos and Ehrlich 2023). The interconnectedness of species within ecosystems swiftly amplifies ecological consequences, a concept Ceballos and Ehrlich (2023) circumscribe using the metaphor “mutilation of the tree of life” (p. 2). In summary, both thousands of scientists and the world’s preeminent global intergovernmental organisation (IGO) with 193 member states recognise the present decline in biodiversity as “unprecedented”, “accelerating”, and “dangerous” (Ripple et al. 2017; IPBES 2019; Tollefson 2019; UN 2019; Ceballos and Ehrlich 2023).

## Epistemological consequences—Science, companionship, and metaphysics

While the mass extinction of species is well documented, its epistemological dimensions remain curiously underacknowledged in the literature. Expressed in simple language, epistemology is the branch of philosophy concerned with knowledge creation. Epistemology examines foundational questions about what knowledge is, how knowledge is acquired, and how knowledge differs from opinions (Everson 1990; Pritchard 2016). Extinction holds vital epistemological implications. Evidently, as species vanish, the scope of what is “knowable” in this world shrinks. Extinction reduces biodiversity and thereby forecloses scientific options for knowledge creation, such as “the discovery of new medicinal resources and pharmaceutical compounds linked to life-saving drugs” (Leal Filho et al. 2024, p. 8). Ceballos and Ehrlich (2023) note this in relation to the recent extinction of the gastric brooding frog that formerly lived in a tiny area of rainforest in Queensland, Australia (Fanning et al. 1982; Tyler et al. 1983):

their extinction to human pressures represents an instance of loss of opportunity for humanity. Their reproduction systems were unique; the females swallowed the newly fertilized eggs and brooded the tadpoles in their stomachs, which were converted into wombs. The frogs were a wonderful model for studying human diseases such as acid reflux and related cancers because the frogs’ stomach acid had to be turned off to protect the brood. But now they are lost to us as experimental models. (Ceballos and Ehrlich 2023, p. 4)

Notably, as the authors point out with support from biodiversity research (Dee et al. 2019), “many species and *genera* with functional ecological traits fundamental for the provision of ecosystem goods and services are rare as the gastric breeding frog” (p. 4).

In addition to foreclosing options for scientific discovery, the epistemological implications of extinction also erode “other trickle-down benefits that may be associated with … human mental health and well-being … birdwatching, and wildlife observation, which foster a meaningful connection between people and nature” (Leal Filho et al. 2024, p. 8). Relatedly, the extinction of species dims the light of knowledge and narrows opportunities for companionship within an ever-shrinking pool of “companion species” (Luetz and Nunn 2023, p. 2037). From an epistemological perspective, the scope of the “knowable” contracts with each vanishing species, erasing opportunities for discovery/erudition and kinship/connection that their existence might have offered. Every species that goes extinct takes with it a unique piece of the world’s knowable mysteries. The extinction of species accelerates the so-called “extinction of experience”—the loss of human–nature interactions (Soga and Gaston 2016).

Significantly, the epistemological implications of extinction also extend beyond science and community to areas of metaphysics and human spirituality. Transcending monotheistic theological orientations, the following discussion examines the consequences of extinction in an interfaith context which recognises that creation stories and myths are not exclusive to the Abrahamic faith traditions (Leeming 1995). Many indigenous worldviews and traditions are deeply spiritual and embrace concepts of a supreme being, spirit, god, or creator/creatrix, often with an emphasis on the interconnectedness between two equally sacred physical and metaphysical worlds (Leeming 1995; LaDuke 2016; Yunkaporta 2019; Luetz 2024). Moreover, some indigenous communities have incorporated elements of Christianity, Islam, or other world religions into their traditions, creating syncretistic beliefs (Jacka 2010; Luetz and Nunn 2021). While some of these beliefs may not fit neatly into Western religious or metaphysical frameworks, they often integrate spirituality, creation myths, and ecology into a cohesive worldview that esteems nature as sacred and indispensable for existential meaning-making (Kealiikanakaoleohaililani and Giardina 2016; LaDuke 2016; Nelson and Shilling 2018; Lykins et al. 2023; Brubacher et al. 2024).

Numerous examples drawn from African philosophies, South American theories, and indigenous worldviews more broadly demonstrate how spiritually grounded frameworks can safeguard the local habitats of endangered species in diverse ecological settings. In Ghana, the sacred status of groves, forests, and totemic species reflects a spiritual ecology that sustains biodiversity through community-based custodianship (Ntiamoa-Baidu 2008; Aniah and Yelfaanibe 2018). Additional African examples illustrate how indigenous societies have lived in harmony with nature, including the Maasai in East Africa (Melubo and Lovelock 2019) and the San peoples of South Africa, whom Lewis-Williams and Pearce (2004) recognise as “the original ecologists” (p. 227). Similarly, in South America, the cosmologies of Amazonian peoples such as the Yanomami and Uitoto integrate ecological knowledge with reverence for non-human life, resisting extractivist conceptions of nature in favour of ecological reciprocity (Simpson 2022; Kopenawa and Albert 2023). These perspectives align with Māori notions of *kaitiakitanga*, where kinship with land and species underpins environmental guardianship (Lockhart et al. 2019), and with Native Hawaiian ethics of sustainability, which emphasise *aloha* *‘āina* (to love the land), *mālama ‘āina* (to care for the land), and *‘ohana* (family) as inclusive concepts that confer a sacred sense of ecological kinship—*‘I ola 'oe, i ola iā‘u nei’* (You live in me, and I live in you) (Kealiikanakaoleohaililani and Giardina 2016, p. 63). Across these worldviews, nature is not a commodity but a sacred relational partner—an outlook that stands in stark contrast to dominant Western paradigms marked by anthropocentrism, ecological domination, and industrial-scale resource extraction (Arbuckle and Konisky 2015; Krenak 2020, 2024).

Applying these understandings to extinction carries important epistemological implications. First, as previously noted, the ongoing process of extinction progressively diminishes the scope of what is “knowable” in the world. This implication will be felt by all humans irrespective of their metaphysical beliefs. Second, as extinction advances, the capacity of the natural world to “reflect” the divine—or embody metaphysical meaning—diminishes. This implication is profoundly relevant for many indigenous communities who esteem nature as sacred and indispensable for existential meaning-making (Kealiikanakaoleohaililani and Giardina 2016; LaDuke 2016; Nelson and Shilling 2018; Nelson 2020; Luetz 2024). It also holds significance for Christian believers who hold that “the heavens declare the glory of God” (Psalm 19: 1) and “the whole earth is full of God’s glory” (Isaiah 6:3). For as extinction unfolds, the earth’s declaration and fullness of God’s “glory” progressively dims and dulls.

A pertinent example of nature’s (vanishing) vibrancy can be found in Australia’s Great Barrier Reef (GBR), designated a UNESCO World Heritage Site in 1981 (UNESCO 2024). Known as the world’s largest living structure and dubbed one of the seven natural wonders of the world, the GBR has been recognised and celebrated for its “superlative natural beauty above and below the water” (Hughes et al. 2017; cf. Climate Council 2024; UNESCO 2024, para. 5). However, the reef is in acute peril from climate change, and “impacts on coral reefs have reached unchartered territory” (Hoegh-Guldberg et al. 2023, p. 1238; Cheng et al. 2024; NOAA 2024). The Climate Council (2024) has warned: “Just as heatwaves on land are getting hotter, longer and more frequent, so too are marine heatwaves, [causing] bleaching events and coral die-offs” (p. 4). Marine heatwaves have been likened to “underwater bushfires” and have reduced the GBR to a new “shadow state” in 2024 (Climate Council 2024, p. 1), with its radiance, health, and vibrancy progressively “fading” (p. 2). The extent of the decline has been so pronounced that scientists studying the reef have reported experiencing “ecological grief” (Conroy 2019, p. 318), described themselves as being “in a state of shock” (p. 318), and, as one researcher emphatically declared, found that “nothing can prepare you for seeing it [in] meltdown” (p. 319). Marshall et al. (2019) have demonstrated a strong link between the dying of the Great Barrier Reef and concomitant declines in human well-being, which the authors call “Reef Grief” (Marshall et al. 2019, p. 579) to signify the “emotional response to the well-documented and publicised degradation of the GBR through coral bleaching and mortality” (p. 585). Tragically, the GBR’s “myriad of brilliant colours” (UNESCO 2024, para. 8) are progressively “fading” (Climate Council 2024, p. 2) as successive bleaching events continue to reduce the GBR to a “shadow state” (Climate Council 2024, p. 1) of its former glory.

There is a large body of literature that supports the view that the spiritual dimension of environmental degradation, extinction, and associated grief, is acutely felt by indigenous and local communities all around the world (Kealiikanakaoleohaililani and Giardina 2016; LaDuke 2016; Nelson 2020; Fernández-Llamazares et al. 2021; Lykins et al. 2023; Brubacher et al. 2024). Understood in this way, the defacing (and desecration) of Australia’s Great Barrier Reef and surrounding islands, has profound spiritual implications for the traditional owners and guardians (Bock et al. 2021; Fischer et al. 2022; Rowland et al. 2024). Multidimensional knowledge is at risk of being lost forever:

Saltwater People, whose Sea Countries include contemporary offshore islands, see these places as remaining intimate aspects of their traditional obligations and custodial identity: the sacred; the spiritual; the creation; the linguistic; the named; the inherited; the known; the restricted; the past and lived present; the sustenance for all life and human livelihoods; the future of current and unborn generations to come. (Bock et al. 2021, p. 316; see also McNiven 2004)

While conceptualisations about nature, identity, spirituality, place, and the divine can differ between communities, there are important metaphysical aspects of human experience that transcend faith traditions. The conservation of biodiversity carries profoundly spiritual dimensions: it preserves what may be known and revered, while extinction dims the light of that knowledge.

Relatedly, in his book *The Vanishing Face of Gaia*, the British scientist James Lovelock (1919–2022) used a helpful metaphor to illustrate that the earth’s self-regulating capacity is progressively “vanishing” (Lovelock 2009). His Gaia theory, though grounded in science, has often been interpreted in spiritual or quasi-religious terms, with some Christians and other faith traditions considering it compatible with belief in divine creation (Rankin 2005; Suárez Müller 2023). His metaphor of the “vanishing face of Gaia” poignantly illustrates the earth incrementally losing its former “face” as ecosystems break down, species die, and climate systems collapse (Lovelock 2009).

This image of a fading planetary face resonates with theological metaphors that frame extinction not merely as ecological loss but also as a spiritual and epistemological rupture. It invites us to consider what is being lost—not only materially, but meaningfully. Viewed through this lens, biodiversity loss becomes not only an ecological crisis but also the silencing of nature’s testimony—a voice through which many have understood the natural world to disclose truths akin to those found in sacred texts. This echoes the theological tradition of reading nature as a book that reveals divine truth alongside scripture (Harrison 2009).

Seen this way, extinction may be understood as epistemologically analogous to “tearing a page out of the Bible” (Merritt 2010, p. 2). If so, how might we characterise the United Nations’ warning (UN 2019) that by the end of this century, “Humans are driving one million species to extinction” (Tollefson 2019, p. 171)? To extend the analogy: if the extinction of each species were equated to erasing a single verse from the canon of scripture, by the end of this century, humanity would have expunged the entire bible more than 28 times.^2^ Relatedly, if past gains in biodiversity are understood as “creation”—as articulated in religious traditions and myths across cultures (Leeming 1995)—then current biodiversity losses might aptly be described as “decreation”, a term McKibben (2005) uses to signify the undoing of “the natural order we found on this earth” (p. 8).

Evidently, humans are, for the most part, completely oblivious to the majority of extinctions occurring around them. Ceballos et al. (2017) note this in relation to most recent extinctions that generate very little public concern “especially because many of those species were obscure and had limited ranges, such as the Catarina pupfish (*Megupsilon aporus*, extinct in 2014), a tiny fish from Mexico” (p. E6089). And yet, extinction implies straightforward epistemological consequences for humans. Simply put, vanished species are no longer knowable, relatable, and appreciable. Christopher Clark, scientist and conservation bioacoustics researcher, has likened the progressive extinction of species to instruments being plucked out of an orchestra:

Everywhere there is life, there is song. The planet is singing—everywhere. But what’s happening is we’re killing the voices … It’s like having a symphony [and] one by one, you just pluck each of the instruments out of the orchestra … and then it’s gone. (Clark 2015; n.p.)

To offer just one example of a disappeared voice from the orchestra of life, Matthews et al. (2024) have documented the extinction of the Kaua'i 'ō'ō (*Moho braccatus*), a Hawaiian songbird that was declared extinct in 2023. The Discovery Channel has posted a recording of its last song for posterity^3^ with the following accompanying commentary: “he is the last male of a species singing for a female who will never come, and now his voice is  gone” (Clark 2015; n.p.).

Although many species may disappear unnoticed by humans—particularly those with limited ranges—the epistemological consequences of extinction are not limited to humans. Within an ecocentric frame of reference (Luetz and Nunn 2023), species are “companion species” (p. 2037) not only to humans but also to each other. Here, then, lies another frequently overlooked aspect of epistemology that transcends humans and needs a holistic lens: extinction spells epistemological consequences by diminishing what companion species may know, need, and/or cherish about each other as fellow sentient beings (Rizzo 2024). Recognising the capacity of animals to have complex emotions, behaviours, and relational experiences, Rizzo (2025) has presented a framework for holistic sustainability that includes all life forms and encapsulates animal sentience and spiritual potential: “Sentience, understood as a spectrum of experiences similar to human consciousness, underpins the view of animals as companion species interwoven within the ecological web of life” (p. 1). Set within an ecocentric frame of reference, extinction implicates “all living beings in the divine narrative” (Rizzo 2024, p. 1) and spells epistemological consequences that extend beyond humans to the more-than-human world.

As shown, the cumulative epistemological consequences of extinction are multifaceted, span the overlapping dimensions of science, community, and metaphysics, and are impossible to quantify. The implications may aptly be described as ubiquitous: the losses are irreversible, cascade across ecosystems, affect all companion species, carry potentially civilisation-scale consequences for humanity, and combine to hasten for all “the depletion of earth’s inspirational and aesthetic resources” (Ceballos et al. 2017, p. E6089). This makes grieving a vital and perhaps inescapable coping mechanism (O’Connor et al. 2008; de Massol de Rebetz 2020) involving humans (Miner 2023), animals (Pokharel et al. 2022; Rizzo 2025), and the wider biosphere: “Ecological grief provides a language for extending grievability beyond human bodies to the whole of our ecosystems” (Bailey and Gerrish 2024, p. 4).

Grieving is an epistemic response to extinction. According to Bailey and Gerrish (2024), ecological grief is the “greatest grief of all” (p. 4). It is acutely experienced by indigenous peoples as a “profound wound” (Morgan et al. 2008, p. 18) and arises from deep and soulful attachment—“it is the deepest love that causes the deepest grief” (p. 18). While indigenous peoples have been bereaved (of their lands) and grieving (biodiversity and land losses) for centuries, scientists studying extinction are only beginning to grapple with the implications (Cunsolo and Ellis 2018; Dudgeon et al. 2024).

Extinction gives rise to a range of complex human emotions, variously characterised as eco-grief, eco-anxiety, and climate anxiety, among other terms and conceptualisations (Conroy 2019; Clayton 2020; de Massol de Rebetz 2020; Hickman 2020; Hickman et al. 2021; Ágoston et al. 2022; Varutti 2024). Significantly, as Conroy (2019) notes, the epistemological and emotional implications of such research are often overlooked: “Although researchers are often on the front lines of ecosystem collapse, few studies have investigated the mental and emotional consequences of such work” (p. 318). In the literature, the emotional toll experienced by scientists represents a very recent emergent field of research interest (Miner 2023; Carrington 2024; Dablander et al. 2024). For example, Schipper et al. (2024) have commented that researchers bear witness

to the extraordinary extent, breadth, magnitude and irreversibility of climate change impacts, and the myriads of associated consequences, that are as yet invisible to many others in society. Moreover, thanks to extraordinary progress in observing, understanding and modelling the climate system and increased capacity for prediction and modelling of socioecological systems, climate researchers now also bear the burden of foresight of seeing what losses and challenges lie ahead and the curse of knowing that these could have been avoided. The feelings that emerge from this aspect of the work of climate researchers will be felt in many ways as individuals, but bringing these to light and making space for emotions and acknowledging them is critical. (p. 1010)

Herein lies an important epistemological consequence of extinction. Studying extinction, including its scope, pace, risks, and corollary consequences, generates knowledge—and concomitant grief about discovered dimensions of loss. Engagement with extinction generates epistemological ripple effects for scientists and other students of biodiversity loss, profoundly shaping their inner worlds as they confront the knowledge of extinctions—past and imminent—and losses that might have been prevented. While much of society remains shielded by varying levels of extinction-ignorance (“I don’t know that I don’t know; I am unaware of my unawareness”), scientists increasingly admit (Conroy 2019; Miner 2023) needing “a safe space to share feelings of anxiety, grief and burnout” (Schipper et al. 2024, p. 1011).

While researchers studying extinction perceive its consequences earlier, more clearly, and more acutely, they are far from alone in grieving its profound losses (Conroy 2019; Miner 2023; Carrington 2024). Mourning is comprehensive and profound—it encompasses a vast and inclusive community of grievers—indigenous communities (Nelson 2020), youth (Hickman et al. 2021), the elderly (Dennis and Stock 2024), scientists (Carrington 2024), activists (Dablander et al. 2024), conservationists (Lees et al. 2020), artists (Bienvenue and Chare 2022), educators (Wilson 2012; Verlie et al. 2021), policymakers (IPBES 2019), laypeople (Law 2019), and members of various faith traditions, including Abrahamic religions (Ouis 1998; Luetz and Leo 2021; Case 2023), and groups adhering to traditional worldview orientations (Brubacher et al. 2024). The experience of grief also extends to the author of this article, who has been recurrently overcome by (irrepressible) tears in both public lectures^4^ and repeatedly during the writing of this article.

Mourning is epistemologically significant. Grieving loss is crucial for emotional healing and psychological well-being (O’Connor et al. 2008). Grieving creates space to process pain, acknowledge the meaning of what has been lost, and to reorient life around what is no longer there. Grieving makes room for reflection, emotional expression, and meaning-making (Varutti 2024). In the context of extinction, grieving may also inspire or invigorate advocacy, activism, and conservation efforts intended to protect what remains (Miner 2023; Dablander et al. 2024; Schipper et al. 2024). By honouring grief, extinction-conscious individuals and communities can cultivate acceptance, purpose, and emotional healing (O’Connor et al. 2008; Clayton 2020). Against this background, Varutti (2024) calls for public mourning as a pathway to existential meaning-making:

The current ecological crisis impels us—we must mourn all losses and we must mourn also for what is about to be lost, for what we don’t know is lost, for the ecosystems we have never known existed or never cared to learn about (p. 562).

As such, grieving is emotional *and* epistemic. It recognises that losses are significant and terminal. In the scientific arena, Cunsolo and Ellis (2018) have characterised ecological grief as a form of knowing-through-feeling, which signals that something irreplaceable is being lost forever. In indigenous settings, grief may be ritualised and expressed communally, functioning as a practice of resisting forgetting and honouring knowledge lost with species or lands (Morgan et al. 2008; Dudgeon et al. 2024). Grief thus becomes a mode of witnessing—a way of knowing extinction—that transcends the technocratic or managerial framings dominant in mainstream conservation science (Lykins et al. 2023). Significantly, grieving extinction also illuminates tensions between dominant Western scientific epistemologies and indigenous or relational epistemologies. In Western science, extinction is typically treated in taxonomic or statistical terms, focusing on quantifiable metrics, population dynamics, and measurable habitat decimation (Anderson et al. 2011; Ceballos et al. 2020). In indigenous epistemologies, extinction is experienced as the loss of reciprocal, spiritual, and kin-based relationships with more-than-human others, sometimes expressed solemnly and ceremonially (McNiven 2004; Kealiikanakaoleohaililani and Giardina 2016). Irrespective of which paradigmatic preference or worldview orientation one may espouse, mourning is a vital human expression of experiencing the epistemological dimensions of extinction. Grieving emerges from an evolving epistemic understanding of the diverse and far-reaching losses permeating the realms of science, community, and metaphysics.

Given the emotional–epistemic entanglements surrounding extinction, a brief critique of the ecosystem services (ES) approach is warranted. This critique highlights the limitations of scientific frameworks in fully capturing the epistemological implications of extinction without engaging cultural, aesthetic, and qualitative modes of understanding. Widely adopted in environmental economics and conservation planning, the ES framework has helped to integrate human-derived benefits from ecosystems into policy and management. Selected benefits include: (1) translating ecological functions into economic metrics, thereby making environmental services visible to policymakers (Costanza et al. 2017); (2) informing decision-making by identifying trade-offs between development and conservation (Daily et al. 2009); (3) linking ecosystems to human well-being, thereby creating narratives conducive to enlisting public engagement (Chan et al. 2012).

Despite these value propositions, the ES approach poses significant epistemological problems and limitations in the context of biodiversity loss and extinction. Critics note, for instance, that (1) commodifying nature risks reducing complex socio-ecological relationships to market-based logics (Schröter et al. 2014); (2) qualitative, non-material, and spiritual values—often crucial to indigenous and local communities—are widely underrepresented (Daniel et al. 2012); (3) the valuation methods themselves are laden with assumptions and uncertainties (Seppelt et al. 2011). Relatedly, Chan et al. (2012) note that “many ecosystem services (co-)produce ‘cultural’ benefits [and embody] non-material values” (p. 8). Ernstson and Sörlin (2013) go even further, arguing that the ES approach is not neutral but tends to universalise, objectify, marketise, and depoliticise ecological values. Its technocratic emphasis on quantitative valuation often marginalises alternative epistemologies—particularly those rooted in indigenous, qualitative, emotional, and spiritual ways of valuing and safeguarding biodiversity. Moreover, ES has a tendency to privilege some actors, namely “experts, consultants, economists, or ecologists” (Ernstson and Sörlin 2013, p. 282), while at the same time silencing and marginalising “voices that are local [and] represent traditional ecological knowledge” (p. 281). By abstracting value from place, history, and spirituality, the ES framework risks marginalising precisely those epistemologies most attuned to biodiversity and species conservation. As extinction erases not only species but also associated cultural and metaphysical knowledge, the limits of the ES approach highlight the need for epistemic pluralism in conservation ethics and policy. Accordingly, conceptions are needed that foreground holistic models of emotional, spiritual, and metaphysical attachments to nature.

## *Sapere aude*—Concluding reflections and future directions

This conceptual paper examined the epistemological implications of species extinction in areas of science, community, and metaphysics, thus contributing theoretical perspectives that transcend purely ecological concerns. The epistemological implications of extinction are considerable: “ecological loss is a radical loss of episteme, the deletion of the very possibility of *ever* knowing and experiencing, a severe restriction to everything that we and future generations might have been and become” (Varutti 2024, p. 557; emphasis original). This article has differentiated the epistemological consequences into three categories (Table 1).

**Table 1:** Overview of extinction epistemologies

Epistemologies of science	*Empiricism: From a scientific perspective,* extinction diminishes what may be known about the world, resulting in an irreversible and permanent loss of opportunities to investigate and harness biodiversity as an inestimable source of scientific knowledge
Epistemologies of community	*Experience: From a cultural perspective,* extinction reduces opportunities for companionship and kin-based relationships with companion species and more-than-human others, diminishing diversity, relationality, culture, and community, and constraining what may be collectively and aesthetically known, shared, and experienced
Epistemologies of metaphysics	*Worldview: From a spiritual perspective,* extinction erodes the capacity of the natural world to reflect the divine or embody soulful ecological mystery, dimming and dulling nature’s sacred and inspirational “glory” and foreclosing avenues to encounter and celebrate the fullness of life and existential meaning

*From a scientific perspective,* species extinction erodes critical knowledge about evolutionary processes and ecological interactions, foreclosing potential biotechnological applications. Each species embodies unique genetic information and ecological roles. Extinction shrinks the gene pool and attendant opportunities to study life, advance medical progress, and innovate solutions to global challenges such as climate change.

*In the realm of community,* extinction disrupts relationships between and among companion species, who play vital roles in areas of identity, mutuality, and emotional well-being. Culturally and aesthetically, extinction diminishes species’ interconnections and the sense of jointly belonging in and to the natural world. Understanding how this evolving cultural severance undermines community points to the affirmation of a mutually shared right to exist.

*From a metaphysical standpoint,* extinction raises profound questions about existence, permanence, and the human story within the web of life. Extinction embodies the ultimate finality and irreversibility of biodiversity loss and points to human moral responsibility in both action and inaction. Extinction also invites humility and a relational epistemology that reverentially recognises the interdependence and inclusion of all beings in shaping and embodying existential meaning.

**Fig. 1:**
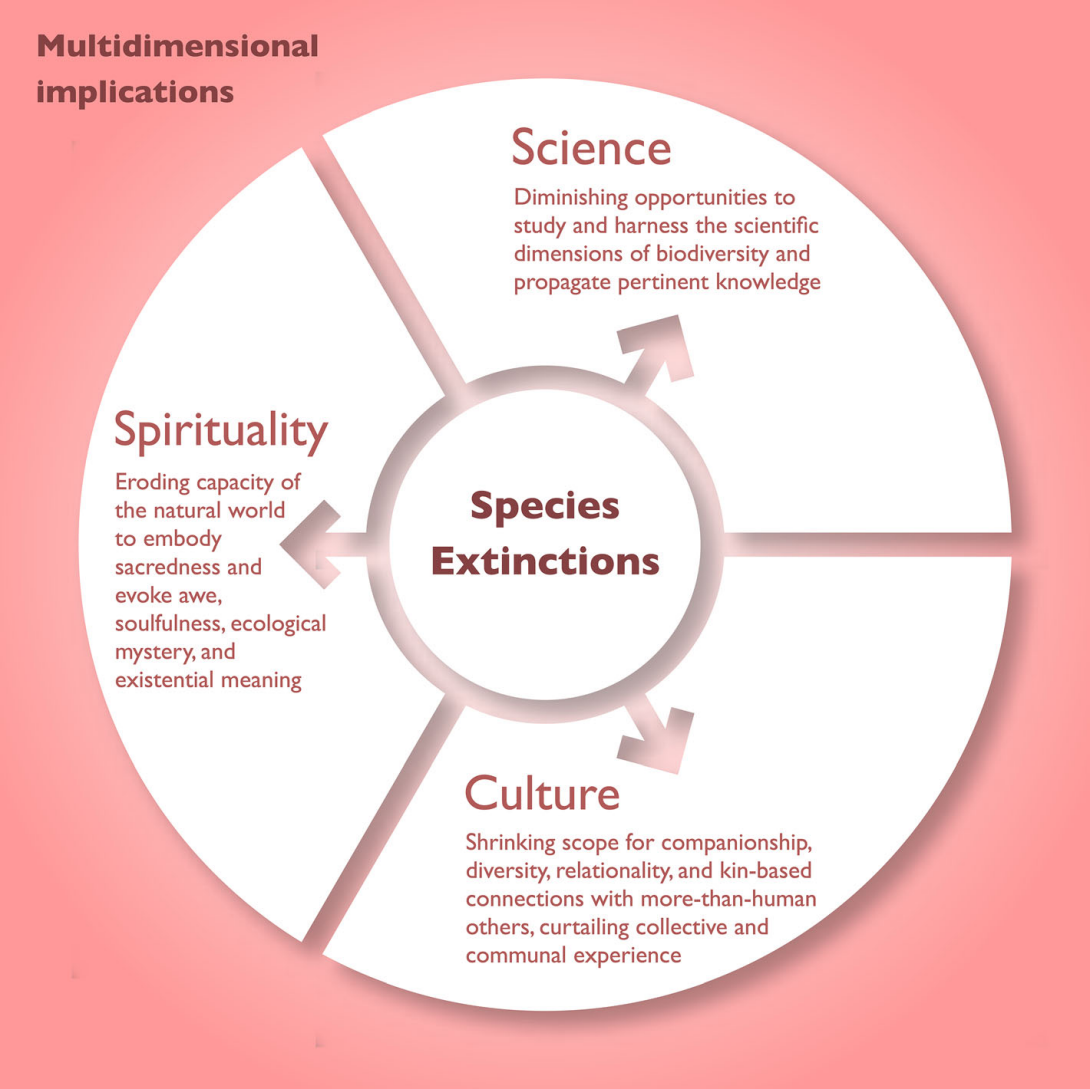
Schematic representation of the triadic epistemological consequences of extinction, encompassing the dimensions of science, community, and metaphysics; the boundaries between these realms are permeable rather than fixed

As illustrated in Fig. 1, extinction dims the light of knowledge across all three realms. Conversely, the epistemological dimensions of extinction position conservation as the unequivocal panacea for safeguarding scientific, cultural, and philosophical or ecotheological richness (Langhammer et al. 2024). To be effective and enduring, conservation must be embedded within a suite of multifaceted strategies. Given that most humans now engage with nature “almost exclusively via the interface of a screen” (Buxton et al. 2021, p. 355), there is a growing need to foster nature-proximate modes of living, learning, and conscientisation (Wilson 2012). There is a broad consensus that proximity to nature nurtures environmentally sympathetic human attitudes and behaviours (Horwitz 1996; Hinds and Sparks 2008; Wilson 2012; Clayton and Myers 2015; Berto et al. 2018; Nelson and Shilling 2018). In response to this, Buxton et al. (2021) have advocated embodied forms of environmental pedagogy—“listening to and learning from and within [nature] as opposed to learning about it from without” (p. 368). With more than half of all humans alive today already residing in cities—so-called “city-zens” (UN-Habitat 2006, p. 6)—and estimates pointing to more than two-thirds of all humans residing in urban conglomerates by 2050 (Ritchie et al. 2024), cultivating nature-proximate affectivity, awareness, and conservation seems no small challenge. Even so, biodiversity conservation plays a vital role in counteracting the “extinction of experience” by maintaining conditions for meaningful engagement with the natural world (Soga and Gaston 2016). Expressed differently, conserving species sustains embodied, affective, and cultural relationships with the environment—thereby safeguarding nature and mitigating the “extinction of experience”.

To conclude, the Latin phrase *sapere aude*—“dare to know”—offers a helpful lens for engaging the epistemological consequences of extinction. Penned by the Roman poet Horace (65–8 BC) and posteriorised in his *Epistulae* (Book 1, Line 40),^5^*sapere aude* was popularised during the Enlightenment by philosopher Immanuel Kant (1724–1804) as a rallying cry for intellectual empowerment, self-determination, and epistemological ingenuity (Kant 1784). Using the phrase *sapere aude* in his essay “Was ist Aufklärung?” (What Is Enlightenment?), Kant (1784) claimed it as the central motif for the Enlightenment era, which profoundly reshaped science, society, and political paradigms (Shapin 1996). Despite being conceived as a cornerstone of modern human development, ongoing critical reflection on the limitations of Enlightenment thinking is warranted. Notably, some of the blame for the current ecological crisis may be placed at the feet of the Enlightenment era and scientific revolution, which

brought about a shift in the perception of nature from humans intrinsically “belonging to it” (and considering themselves as an integral part of it) to humans impartially “observing it” (and somehow viewing themselves as being on the outside and separate from it). (Luetz and Nunn 2023, p. 2037)

The ensuing paradigm shift nurtured the perception of “subject/object duality” (Nelson 2020, n.p.), intensifying the sense of separation—or even estrangement—between humans and nature, and hastening the progressive commodification of the natural world. Amid the ongoing crisis of species extinction, *sapere aude* may be rediscovered, reappropriated, and reclaimed—not merely as a philosophical imperative of a bygone era, but as a contemporary rallying cry for the critical conservation of knowledge itself. Embodying a spirit of curiosity, inquiry, and responsibility in the pursuit of knowledge, *sapere aude* may be reimagined as an epistemologically resonant concept for transcending paralysis—where “collective inaction is tantamount to complicity” (Varutti 2024, p. 562). The disappearance of species erases unique genetic, cultural, and metaphysical resources and challenges humans to seek wisdom—*sapere aude*—in the face of irreversible and inestimable loss. This implies various practical and moral questions, which are raised here to inform future research directions:

Can conservation efforts be reframed as epistemological projects aimed at safeguarding not just biological entities but also the dimensions of knowledge they embody? How should the risk of epistemic loss reshape conservation priorities and scientific research agendas? (Langhammer et al. 2024) How does extinction disproportionately affect marginalised communities, including indigenous peoples, whose knowledge systems are often closely tied to threatened species and ecosystems? What are the implications of privileging Western scientific perspectives over traditional ecological knowledge in the context of extinction? How can obstacles be overcome? (Leal Filho et al. 2025) How may planetary-scale conservation be reshaped by the cosmologies of indigenous peoples, wherein nature tends to be “revered as proximate, sacred, or even en-spirited, [in contrast] to the quasi-ubiquitous anthropocentric view that rationally and dispassionately—and perhaps even condescendingly—considers nature as existing predominantly for the sake and benefit of humans”? (Luetz 2024, p. 732) What are the responsibilities towards future generations in preserving knowledge tied to biodiversity? Can the (growing) human community of the bereaved be mobilised to come together and collaborate as a unified conservation coalition? What role do public tears and mourning play in expressing grief over ecological loss, and (how) might such emotions be harnessed for conscientisation and conservation activism? (Burton-Christie 2011; Varutti 2024) Is the extinction of every species always and necessarily a tragedy—thinking, for example, of the dinosaurs—even though there is an epistemological loss each time? What are the roles of biobanking (cryopreservation) and de-extinction science (resurrection biology) in conservation planning, and what are the ethical dimensions and limitations? (Moore and Nelson 2010; Sayre 2017; Wienhues et al. 2023; Daly et al. 2024).

Finally, while this paper adopted a balanced, inclusive, and worldview-neutral stance on extinction and epistemology, future research may more explicitly explore the distinctive contributions of specific religious traditions, as exemplified below in reference to Christianity. Focusing more deeply and uniquely on Christianity, for example, is warranted for historical, socio-cultural, and epistemological reasons. Although inclusive engagement with other religions, worldviews, and indigenous spiritualities remains essential (as documented in this article), there are at least four specific rationales for paying particular attention to Christianity.

First, Christianity has been a foundational historical influence on Western epistemologies, shaping notions of nature, humanity, and nature discourses. This legacy includes anthropocentric hierarchies rooted in certain Christian theologies (e.g., dominion in Genesis 1: 28) that have historically underpinned exploitative approaches to the natural world (Luetz and Leo 2021). Lyn White (1967) famously described Christianity, at least in its Western form, as “the most anthropocentric religion the world has ever seen” (p. 1205). More to the point, modern “science and technology have grown out of Christian attitudes” (White 1967, p. 1206). This legacy makes Christianity a crucial interlocutor in rethinking epistemologies that either contributed to or could transform dominant understandings of nature, environmentalism, and extinction (Edvardsson Björnberg and Karlsson 2022).

Second, Christianity wields institutional and political influence in global environmental arenas, particularly through the Catholic Church and major Protestant bodies such as the Lausanne Movement and World Evangelical Alliance Creation Care network (LWCCN)^6^ and A Rocha,^7^ among others. This gives Christianity the capacity to shape discourse and policy, such as through Pope Francis’s 2015 encyclical *Laudato Si’*, which reframes ecological degradation and species extinction as moral and spiritual crises (Pope Francis 2015). Conversely, this considerable influence has also rendered Christianity a formidable force that self-professed adherents have co-opted to peddle climate science denial and extractivist agendas (Arbuckle and Konisky 2015; Edvardsson Björnberg et al. 2017, 2020).

Third, Christian traditions offer deep theological and ethical resources (e.g., doctrines of creation, incarnation, stewardship, and eschatology) that can be mobilised to reframe human–nonhuman relationships and contest extractivist logics. Scholars such as Sallie McFague (2008), Jürgen Moltmann (2010, 2012), and Elizabeth Johnson (2014) have called for ecotheological shifts that reimagine creation as interconnected and sacred. Relatedly, Christian eschatology uniquely engages with themes of end-times, hope, and renewal—offering a distinctive metaphysical lens through which to reflect on extinction, finitude, and ecological grief. This positionality can foster profound ethical and spiritual engagement with extinction and existential meaning beyond technocratic, instrumental, or utilitarian paradigms (Luetz et al. 2018; Bangert 2021).

Fourth, Christian mission history is intertwined with colonial projects that displaced indigenous ontologies and contributed to environmental and cultural destruction. In the words of White (1967), “Christianity bears a huge burden of guilt” (p. 1206). Addressing this complicity can open space for theological repentance, reform, and solidarity with decolonial and indigenous knowledge systems (Budden 2009). Relatedly, focusing on Christianity vis-à-vis other religions may stimulate interfaith and intercultural dialogues that are critical for epistemic justice. Relevant interfaith engagement may encourage reconciliation with indigenous cosmologies that have long held relational ontologies and emphasised inter-species kinship, often suppressed under Christian-colonial worldviews in invaded space (Budden 2009).

An initial attempt to apply some of the perspectives developed in this article within a Christian education context has been made (Luetz 2025). Future research could more systematically explore the distinctive contributions of particular Christian traditions. Crucially, Christian communities—like other religious groups—are not monolithic; they require careful differentiation, as they can play diverse and multifaceted roles in advancing sustainable futures (Koehrsen and Ives 2025). Investigating the varied and evolving forms of religious engagement with extinction and epistemology therefore presents fertile directions for future interdisciplinary inquiry.

In summary, the above questions, opportunities, and research directions underscore the need for holistic, interdisciplinary, and enduring efforts that integrate research on science, ethics, culture, faith, and philosophy to fully map and understand the epistemological consequences of extinction. By engaging these and other epistemological questions and challenges, the (human) species may not only honour (companion) species already lost, but also deepen its resolve to safeguard the (remaining) interconnected mysteries of life from further erasure. Recognising the irreplaceable value of life in all its forms can inspire and uphold strategies that honour both tangible (physical) and intangible (metaphysical) dimensions and contributions. In this sense, conserving biodiversity mitigates the epistemological losses linked to extinction by preserving scientific, aesthetic, cultural, and inspirational resources; sustaining the shared knowledge, experiences, and relationships of companion species; and safeguarding what may (yet) be known, revered, or worshipped in nature, the divine, and the metaphysical.

## Data Availability

The author declares that all data supporting this study are available within the paper.

## Footnotes

1 *Genera* (plural of *genus*) are taxonomic categories that group together closely related species; they are positioned above species and below family in the biological classification hierarchy. Members of a *genus* typically share common traits and a common evolutionary ancestor. For example, *Panthera* includes lions (*Panthera leo*), tigers (*Panthera tigris*), and leopards (*Panthera pardus*) (Tudge 2000).

2 This number may increase to 43, depending on the bible version used. Note that variations in verse counts arise from differences in canon, translation practices, and versification systems across religious traditions and bible editions. The following figures are broadly representative: Protestant bible (e.g., NIV or KJV): 66 books/ ~ 31,000 verses; Catholic bible: 73 books/ ~ 35,000 verses (incl. seven deutero-canonical books); Hebrew bible (Tanakh): 24 books/ ~ 23,000 verses (varying divisions and versification).

3 https://youtu.be/tRaC2Rx3BVY?feature=shared&t=57 see also: https://youtu.be/5THqAY3u5oY.

4 One such instance occurred during an invited keynote address at the *Educating for Hope* symposium, held in Sydney on 9 May 2025—where unbidden moments of visible emotion bore public witness to the author’s grief while speaking about biodiversity loss (documented in Luetz 2025). Such acts of public grieving resonate with Varutti’s (2024) insight that mourning can render extinction both commemorative and emotionally intelligible to wider publics. Likewise, Burton-Christie (2011) interprets public tears as an embodied expression of ecological compassion and a subtle form of resistance to environmental degradation and/or human indifference.

5 https://www.thelatinlibrary.com/horace/epist1.shtml.

6 http://lwccn.com/.

7 https://arocha.org/.
